# Elevated percentage of HLA-DR^+^ and ICAM-1^+^ conjunctival epithelial cells in active Graves’ orbitopathy

**DOI:** 10.1007/s00417-014-2580-z

**Published:** 2014-02-23

**Authors:** P. Pawlowski, J. Mysliwiec, M. Mrugacz, J. Zak, A. Bakunowicz-Lazarczyk, R. Rejdak, J. Wysocka, M. Gorska

**Affiliations:** 1 Department of Pediatric Ophthalmology with Strabismus Treatment Unit, Medical University of Bialystok, 17 Waszyngtona Str, 15-274 Bialystok, Poland; 2 Department of Ophthalmology, Provincial Combined Hospital in Bialystok, Bialystok, Poland; 3 Department of Nuclear Medicine, Medical University of Bialystok, Bialystok, Poland; 4 Department of Pediatric Laboratory Diagnostics, Medical University of Bialystok, Bialystok, Poland; 5 Department of General Ophthalmology, Medical University of Lublin, Lublin, Poland; 6 Department of Experimental Pharmacology, Medical Research Centre, Polish Academy of Sciences, Warsaw, Poland; 7 Department of Endocrinology, Diabetology and Internal Diseases, Medical University of Bialystok, Bialystok, Poland

**Keywords:** HLA-DR, ICAM-1, Graves’ orbitopathy, Conjunctival epithelial cells (CEC), Topical inflammation

## Abstract

**Background:**

To evaluate if conjunctival epithelial cells’ expression of HLA-DR and ICAM-1 could be helpful as early topical markers of inflammation in Graves’ orbitopathy (GO).

**Methods:**

The ocular examination evaluated a clinical activity score (CAS) by assessment of clinical features, (e.g., eyelid or conjunctival inflammation, lid width, lid closure, proptosis, ocular motility). Conjunctival epithelial cell specimens for flow-cytometric evaluations of ICAM-I and HLADR expression were collected by impression cytology from ten eyes with active GO (CAS ≥ 4 and duration ≤ 12 months), from 15 eyes with Graves’ disease (GD) without active GO (CAS 0–2) and from 15 normal specimens without any ocular disorders.

**Results:**

The percentage of HLA-DR + conjunctival epithelial cells was significantly elevated in patients with active GO comparing to GD without active GO and healthy controls, 10.7 % (8.5–17.7) and 7.78 % (3.92–10.1) (*p* < 0.05) vs. control 4.89 % (3.5–5.5) (*p* < 0.005), respectively. The expression of ICAM − 1+ conjunctival epithelial cells was greater only in patients with GO vs. controls, 5.5 % (4.8–7.03) and 1.46 % (0.69–2.51) (*p* < 0.005), respectively.

**Conclusion:**

The percentage of HLA-DR^+^ and ICAM-1^+^ conjunctival epithelial cells in patients with the active GO may serve as a topical inflammation marker in Graves’ orbitopathy.

## Introduction

Development of flow cytometric analysis of cytologic specimens provides a new, sensitive and objective tool for exploring conjunctival pathology [1]. Conjunctival epithelium expression of HLA-DR (human leukocyte antigen-DR) and ICAM-1 (intercellular adhesion molecule-1) have been shown to be correlated with some topical inflammatory processes like allergic conjunctivitis, pterygium, or keratoconjunctivitis sicca (KCS) in cystic fibrosis [2–6].

During inflammation, the expression of endothelial and epithelial ICAM-1 increases [7]. ICAM-1 attracts leukocytes to the conjunctival surface and holds them there [8]. Conjunctival expression of HLA-DR is normally restricted to resident dendritic cells, but induction of class II antigens in epithelial cells has consistently been shown to be associated with conjunctival inflammatory reactions [5, 7, 8]. Graves’ orbitopathy (GO) is known to be an autoimmune process leading to inflammation of the orbital tissue [9]. In addition, almost all patients with GO show signs of ocular surface damage [10]. Hence, detection of HLA-DR and ICAM-1 molecules on the conjunctiva may suggest activity of the immunopathological process underlying Graves' orbitopathy which should be considered as an indication of intensive systemic immunosuppression.

The objective of this study was to evaluate the expression of ICAM-1 and HLA-DR in conjunctival epithelial cells by flow cytometry in patients with active GO and Graves disease (GD) without active GO, in order to estimate if the conjunctival expression of these molecules could serve as topical markers of ongoing inflammation in the active GO.

## Materials and methods

### Patients and controls

Conjunctival impression cytology was obtained from ten eyes (ten patients) (eight females, two males) a mean age of 35.9 ± 9.9 years (range 26–46 years) with active GO (CAS ≥ 4), from 15 eyes (15 patients)(12 females, three males) with a mean age of 36.5 ± 11.5 years (range 25–48 years) with GD without signs of active GO, recruited from the Department of Endocrinology, Diabetology and Internal Diseases of Medical University of Bialystok. Fifteen impression cytology specimens were obtained from 15 eyes of ten healthy volunteers (eight females, two males) a mean age of 34.5 ± 12 years (range 22.5–47 years) to serve as a control.

The study was carried out with approval from the Ethics Committee of the Medical University of Bialystok, in accordance with the guidelines of the Helsinki Declaration. All patients and control persons gave their informed consent prior to inclusion in this study.

### Clinical examination

The ocular examination evaluated the best corrected visual acuity (Snellen charts), colour vision (Ishihara charts), intraocular pressure (applanation tonometry) and exophthalmus. The GO clinical activity score included measurements of lid aperture, marginal reflex distance, the presence of eyelid retraction, eyelid or conjunctival inflammation, biomicroscopic examination of the anterior segment, indirect ophthalmoscopy, and ocular motility.

### Grouping of patients

Signs of GO in the eye were classified according to the Clinical Activity Score (CAS) classification and NOSPECS amended by EUGOGO [11]:

1. *active GO* – CAS equal to or greater than 4 and/or NOSPECS equal to or greater than 5 (marked symptoms of GO); Manifestation of GO ≤ 12 months.
2. *GD without signs of active GO* – CAS less than 4 and NOSPECS less than 5 (mild symptoms of GO) or no signs of ophthalmopathy.

#### Exclusion criteria

Patients with allergic conjunctivitis, dry eye, blepharitis, uveitis, infective conjunctivitis or keratitis were excluded from the study. Also, patients with a history of ocular surgery within the last three months or patients with any other ocular disease that might alter the markers to be measured were excluded from the study. Patients receiving topical corticosteroids or anti-inflammatory treatment within the last month or patients who had radioiodine therapy or with any immune system disease were also excluded.

### Impression cytology of conjunctival epithelial cells and flow cytometric assessment

**Fig. 1:**
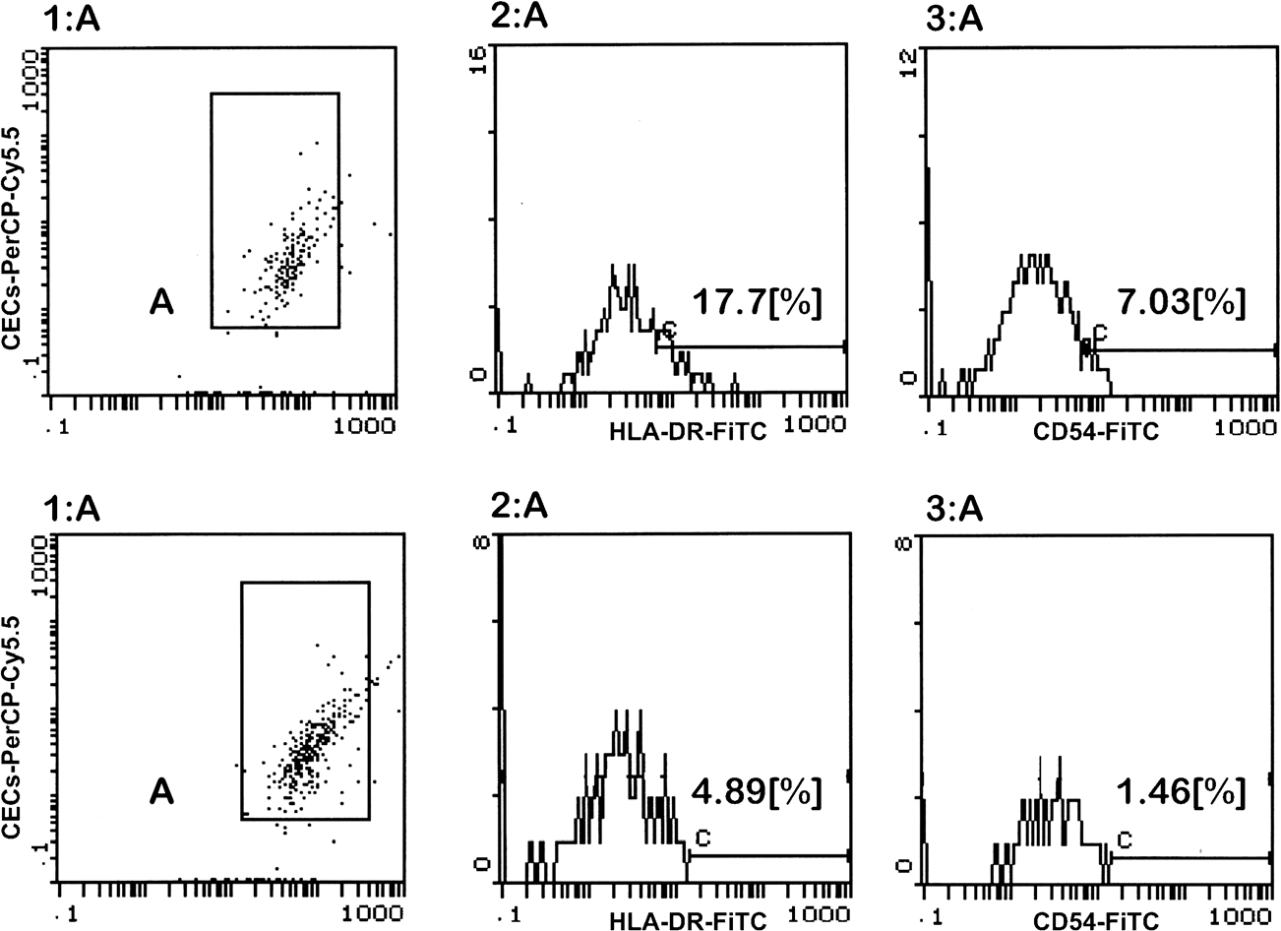
Flow cytometric scatter-plots: first row of gates demonstrates a specimen from active GO patient, second row shows a healthy control. 1A) displays the CECs gating, 2A) HLADR^+^ CECs’ cells counting, 3A) ICAM-1^+^ CECs’ counting

After topical anaesthesia with 1–2 drops of 0.04 % oxybuprocaine, two parts 13 × 6.5 mm in size (polyethersulfone filter, 0.20-μm pores, Supor, Gelman Sciences, Ann Arbor, MI) were applied onto the superior and superiortemporal bulbar conjunctiva without exerting any pressure. All membranes were immediately dipped into tubes containing 1.5 ml of cold phosphate-buffered saline (PBS) with fixative (0.05 % paraformaldehyde). Cells were extracted by gentle agitation for 20 min and centrifuged (1,800 rpm, 5 min). For each sample 2,000–5,000 cells were analyzed and their fluorescence output was displayed on a log scale. Monoclonal antibodies with fluorochromes: CD54-FITC (mouse IgG1, clone 6.5B5) and antibodies against class II antigen HLA-DR-FITC and EpCAM-PerCP-Cy5.5 (mouse IgG1, clone EBA-1) antibodies were purchased from DakoCytomation (Copenhagen, Denmark) and Becton Dickinson (Mountain View, CA, USA), respectively. A nonimmune mouse IgG1-FITC (clone DAK-G01) was used as a negative isotypic control (DakoCytomation). Direct immunostainings were performed with monoclonal antibodies for 30 min at 4 º C. A two parameter analysis was performed to determine the expression of ICAM-1 and HLA-DR molecules on EpCAM positive cells (conjunctival epithelial cells). The flow cytometric analysis was performed with a Coulter Epics-XL flow cytometer (Beckman Coulter Corporation, Miami, FL, USA). The results are expressed as a percentage of positive cells (Fig. 1).

### Statistical analysis

The patients'characteristic data and ocular assessments are given as mean ± SD or median with range (in brackets). The median and quartile values of the ICAM-1^+^ and HLADR^+^ conjunctival epithelial cells were presented. The statistical significance of the evaluated CEC subsets’ expression was measured by the U Mann–Whitney test. A p value of less than 0.05 was considered statistically significant. All data were performed using Statistica 9.0 (StatSoft ®, Tulsa, OK, U.S.A.).

## Results

### Clinical features of the study groups

All results obtained for GO dependent alterations have been summerized in Table 1.

**Table 1:** Comparison of clinical features of active GO and GD patients without active GO. Date are presented as mean ± SD, or median value and max and min (in the brackets). F: female, M: male

	*Active GO*	*GD without active GO*	*p value*
Number of specimens	10 eyes	15 eyes	
Age	35.9 ± 9.9	36.5 ± 11.5	0.534
Sex	8 F/2 M	12 F/3 M	
Time of GO (months)	5 (2–9)	8 (0–18)	
Clinical activity score (CAS)	6 (4–8)	1 (0–2)	
NOSPECS (Disease severity classification)	6 (5–7)	2 (0–4)	
Proptosis (mm)	22 (17–26)	18 (14–22)	0.08
Palpebral fissure width (mm)	12.5 (9–14)	11 (9–13)	0.20

### The expression of HLA-DR and ICAM-1 on CECs

**Fig. 2:**
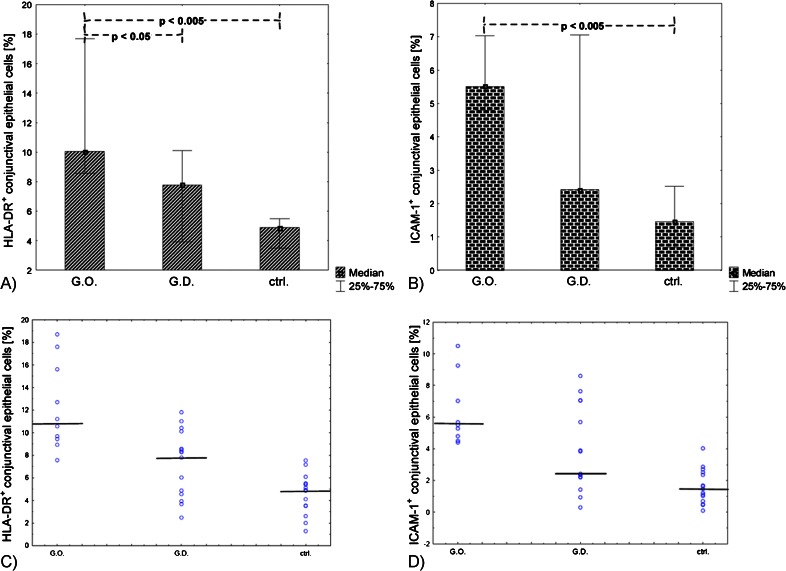
The median and the interquartile range of (**a**) HLA-DR+, and (**b**) ICAM-1+ conjunctival epithelial cells’ percentage in patient with active GO, GD without active GO and controls (whiskers display the interquartile range). (**c**) scatter plots of HLA-DR + CEC, and (**d**) scatter plots of ICAM-1+ CEC in patients with active GO, GD without active GO and controls (bold line displays median value)

The percentage of HLA-DR^+^ conjunctival epithelial cells was significantly elevated in patients with active GO comparing to GD without active GO and versus healthy controls (*p* < 0.005 and *p* < 0.,05 respectively) (Table 2, Fig. 2). Moreover the median percentage of HLA-DR^+^ CEC in active GO (10.7 % (8.5–17.7) was significantly greater than in patients with GD without active GO (7.78 % (3.92–10.1) (*p* < 0.05)). The median percentage of HLA-DR + epithelial cells in active GO was two-folds higher than in healthy controls (10.7 % (8.5–17.7) vs. 4.89 % (3.5–5.5)) (Table 2, Fig. 2).

**Table 2:** The median and quartile values of the percentage of HLADR^+^ and ICAM-1^+^ conjunctival epithelial cells in the studied groups

*Parameter*	*Active GO*	*GD without active GO*	*Ctrl*.
HLA-DR %	10.7** (8.5–17.7)	7.78* (3.92–10.1)	4.89 (3.5–5.5)
ICAM-1 %	5.5** (4.8–7.03)	2.42 (0.94–7.05)	1.46 (0.69–2.51)

**p < 0.005 active GO vs. controls

*p < 0.05 active GO vs. GD without active GO for HLA-DR^+^ CECs

The statistical analysis of ICAM-1^+^ conjunctival epithelial cells revealed the elevated percentage of these cells in patients with active GO vs. controls (5.5 % (4.8–7.03) and 1.46 % (0.69–2.51), respectively)(*p* < 0.005) (Table 2, Fig. 2). In patients with GD without active GO the expression of ICAM-1^+^ CECs was not increased comparing to control (2.42 % (0.94–7.05) (Table 2).

## Discussion

It was shown that incomplete blink, increased proptosis and greater palpebral fissure width in GO accelerates tear evaporation, which increases the fluid’s osmolarity and results in ocular surface damage [10]. Recently, it has been found that inflammation plays a key role in ocular surface damage of GO [12–15]. The increased concentrations of tears’ inflammatory cytokines (IL-1β, IL- 6) and chemokine IL-8 in patients with active vs. inactive TAO (thyroid associated ophthalmopathy) supports this thesis [16]. Gupta suggested that TAO was a potential (occult) cause of inflammatory ocular surface disease with dry eye symptoms [12].

In our previous study, serum L-selectin and ICAM-1 were found to be elevated in patients with active GO, suggesting enhanced T cell recruitment [17]. In accordance with that, we have shown an increased percentage of L-selectin + and ICAM-1+ peripheral blood CD4+/CD8+ T cells in active GO [18]. Since the orbital tissues are infiltrated with inflammatory and immune cells in GO [10], the ICAM-1 and the HLA-DR expression on the ocular surface may serve as markers of a inflammatory status in GO. ICAM-1 plays a central role not only in T lymphocyte activation but also T cell trafficking at the inflammatory sites, which may result in amplification of the cellular immune process in active GO (Bahn 2003). The presence of HLA-DR renders conjunctival epithelial cells capable of presenting antigens, as investigated by impression cytology, which could be an indirect indicator of the involvement of the Th1 subset [19]. Not only the increased percentage of HLA-DR^+^ CECs in patients with active GO vs. controls, but also vs. GD patients without active GO may suggests the local activation in these patients is due to different immunological pathways. It has been shown that the Th1 profile predominates in the early active phase of GO [20], which can be attributed to the local HLA-DR engagement. In agreement with that, our study showed a significantly higher percentage of HLA-DR + CECs in patients with active GO than in patients without active GO.

Kulig et al., found that the serum levels of ICAM-1 seem to be a more sensitive marker than MRI in the assessment of the activity of GO [21]. Our study is one of the few that show the topical activation markers may be useful for diagnosing active GO. Further studies should be designed to elucidate the role of conjunctival epithelial cells’ immunophenotyping in monitoring the effects of systemic immunomodulatory treatment in active GO [17].

In conclusion, our results might be a good hint that the HLA-DR + CECs and ICAM-1+ CECs immunophenotyping could be a topical inflammation marker in active GO.
